# Protein Matters and Proteins Matter: Proteomics and Peptidomics in Nutrition and Health

**DOI:** 10.3390/nu15122762

**Published:** 2023-06-15

**Authors:** Martin Kussmann

**Affiliations:** 1 Kompetenzzentrum für Ernährung (KErn), 85354 Freising, Germany; martin.kussmann@kern.bayern.de or martin@kussmann-biotech.com or martin@kussmann.ch; Tel.: +41-79-471-64-59; 2 Kussmann Biotech GmbH, 59394 Nordkirchen, Germany

Protein matters and proteins matter in nutrition and health—why?

Protein is one of the three macronutrients for the human body, providing amino acids and building blocks for cell, tissue, and organ maintenance and turnover. Proteins are the “molecular robots” in living systems implied in controlling, tuning, and executing molecular functions, from gene expression to metabolism.

Nutrition and proteomics form a natural merger of health science with bioanalytical technology. Nutrition has the strongest life-long impact on human health, and the science has evolved from elucidating macro- and micronutrient requirements in populations to optimizing diets and delivering functional foods and ingredients for personalized nutrition, all to maintain health, prevent (chronic) disease, and enhance performance and wellbeing.

Mass spectrometry-based proteomics and peptidomics are the leading platforms for the comprehensive analysis of proteins and peptides to assess food composition, quality, authenticity, and safety, and to identify biomarkers for nutritional predisposition and intervention. For this Special Issue, studies are collected on cutting-edge science about proteomics and peptidomics technology, including bioinformatics, and their deployment for (a) the analysis of food composition, quality, authenticity, and safety; (b) the identification and characterization of directional food–health relationships; and (c) the discovery and characterization of bioactives with specific health benefits, thereby leveraging food proteins beyond their purely nutritional value.

Following this scope and logic, I herewith shortly introduce the contributions and the teams behind the work: Sarah Brajkovic, Claus Schwechheimer, Bernhard Kuster, and collaborators at the Technical University, Munich, and the Leibniz University, Leipzig, Germany, describe the technology and methods needed for “large-scale proteomics in crop plants”—the proteomes that feed the world. Jacqueline Monteiro from the University São Paolo, Brazil, and Jim Kaput et al. from Vydiant Inc., Madison, WI, USA, identified and analyzed “topic clusters in a nutri-, food-, and diet-proteomic corpus using machine reading”. I report on the “prediction, discovery, and characterization of plant- and food-derived health-beneficial bioactive peptides”. Salvatore Foti et al. from the University Catania, Italy, introduce “a manually curated database of the metabolic proteins of *Triticum aestivum*. Ana G. Abril and Mónica Carrera et al. from the University of Santiago de Compostela, and the Spanish National Research Council, Vigo, Spain, present a “proteomic characterization of food-derived anti-allergic and anti-inflammatory peptides”. Mengzhen Hao, Huilian Che, and colleagues from the China Agriculture University, Beijing, identified “allergens in Pitaya seeds using proteomics and immunoinformatics”. Finally, Federica Farabegoli, Ignacio Ortea, and Celina Costas et al. from various research institutions across Spain explored “anti-inflammatory effects of inulin in a murine macrophage cell model by transcriptomics and proteomics”.

I hope you enjoy reading these articles, thereby receiving an update on a fascinating field of life science. I would like to thank all contributors, among who are many long-term colleagues and friends, and the *Nutrients* editorial team for having made this Special Issue possible and bringing it to life.

